# U.S. Federal and State Medicaid Spending: Health Policy Patterns by Political Party Leadership and Census Demographics

**DOI:** 10.3390/ijerph22071074

**Published:** 2025-07-04

**Authors:** Jamaji C. Nwanaji-Enwerem, Pamaji Nwanaji-Enwerem

**Affiliations:** 1 Center for Health Justice, Department of Emergency Medicine, Perelman School of Medicine, University of Pennsylvania, Philadelphia, PA 19104, USA; 2 Center of Excellence in Environmental Toxicology, Department of Emergency Medicine, Perelman School of Medicine, University of Pennsylvania, Philadelphia, PA 19104, USA; 3 Department of Business and Entrepreneurship, Barber-Scotia College, Concord, NC 28025, USA

**Keywords:** congress, health policy, insurance, political party, reform, Medicaid

## Abstract

Medicaid is a vital public health program, serving over 70 million Americans from many backgrounds. Understanding how Medicaid spending varies by political leadership and demographic factors can inform policy discussions and advocacy efforts. We conducted a cross-sectional descriptive analysis of state Medicaid spending using publicly available data. Our findings show that individuals identifying as White comprise the largest single racial group of Medicaid beneficiaries both nationally and in most states. While the 2020 Census Diversity Index correlated strongly with total Medicaid spending, no significant association was found with per enrollee spending or the federal share of Medicaid funding. States led by Democrats had higher total Medicaid spending when compared to Republican-led states. However, Republican-led states received a larger proportion of federal Medicaid funding. Among political leadership levels, Senate representation showed the strongest relationship with Medicaid spending trends compared to gubernatorial leadership and presidential voting history. In conclusion, we demonstrate that Medicaid spending impacts all racial groups and both major political parties. However, funding structures and political representation reflect distinct spending patterns. Given the evolving demographic and political landscape, ongoing policy discussions should ensure that Medicaid remains a public health program that remains effective at safeguarding human health.

## 1. Introduction

Since its creation through the Social Security Amendments of 1965, Medicaid has literally served as a lifeline for some of America’s most vulnerable people [1]. With over 72 million Americans enrolled, Medicaid is the United States’ (U.S.) largest public health insurance program—helping to provide healthcare to low-income adults, children, and people with disabilities [2]. Inpatient hospital admissions, laboratory/radiology testing, nursing facility care, family planning, tobacco cessation counseling for pregnant women, and access to nurse midwives are among some of the federally mandated services that Medi-caid covers. In some states, Medicaid may cover additional services, including dental care, physical therapy, prescription medications, eyeglasses, prosthetics, and hospice [3]. Given its large impact and support of over 20% of the U.S. population, it is not surprising that Medicaid comes with some notable financial costs. Importantly, Medicaid is structured as a joint federal and state program with funds being drawn from both levels of government [1]. State Medicaid expenditures are matched by federal funds using formulas that consider factors like per capita income among states. In 2022, the federal government spent USD 592 billion on Medicaid, covering 71% of the program’s costs [4].

Because of Medicaid’s financial cost, there continue to be conversations on ways to improve the program and make it more efficient [5]. However, these conversations are met with concerns about what Medicaid cuts would mean for the health of its enrollees. Notably, cuts in Medicaid implemented in the 1980s were associated with clinically meaningful deteriorations of enrollee health as soon as six months after losing coverage [6,7]. Thus, it remains important to center any conversations about Medicaid reform on maintaining and improving human health. Still, recent dialogue in the 119th Congress (convened on 3 January 2025) has remained largely partisan, reflecting previously observed political trends. For example, polling by the Kaiser Family Foundation highlights persistent ideological divides in how Americans perceive Medicaid. While a majority of Democrats (79%) and Independents (60%) view Medicaid primarily as a health insurance program, most Republicans (54%) see it as a government welfare program [8]. In an effort to move beyond often abstract ideological and partisan debates and to refocus attention on the real-world human impact of Medicaid reform, we use publicly available data to examine the state-level diversity of Medicaid beneficiaries and illustrate the program’s broad and tangible benefits to many Americans. Additionally, to further promote unity on this issue in a divisive political climate, we describe state-level patterns of Medicaid spending in the context of political party leadership. More specifically, we examine patterns of total Medicaid spending, per enrollee Medicaid spending, and the federal share of Medicaid spending (also known as the federal medical assistance percentage-FMAP) in each state by whether each state assigned their electoral college votes to a Democrat or Republican candidate in the presidential election and if the state is represented by Democrats or Republicans on the Senate and gubernatorial levels. Once again, our goal is to demonstrate that Medicaid reform is not merely a partisan issue, but one that affects the health and well-being of Americans across the political spectrum.

## 2. Methods

### 2.1. Data Sources

We conducted this analysis using publicly available data. State-level data for the 2020 diversity index was downloaded from the U.S. Census Bureau website and was used as our measure of state racial/ethnic diversity [9]. The diversity index can range from 0% to 100% and describes the chance that two people, when chosen at random, will be from different race/ethnicity groups. A diversity index of 0% means that everyone in a population is of the same race/ethnicity, while values closer to 100% indicate greater diversity. The U.S. Census Bureau reports that in 2020, the U.S. had a national diversity index of 61.1%. State-level total Medicaid spending, FMAP, and per enrollee Medicaid spending data were downloaded from the Medicaid and CHIP Payment and Access Commission (MACPAC) website [10,11]. The per enrollee data were from 2022, while the total spending and FMAP spending data were from fiscal year 2023. State-level race/ethnicity data on the distribution of people ages 0–64 with Medicaid for 2023 was downloaded from the Kaiser Family Foundation website [12]. Data on congressional Senate representatives and their political parties was available from congress.gov. Data on governors was available through Multi-State and “The Book of States” [13,14,15]. Given that the District of Columbia does not have a governor, we used the political party of their chief executive (mayor) instead. State-level presidential electoral college results were downloaded from archives.gov [16].

### 2.2. Statistical Analysis

Spearman correlations were used to examine relationships of the census diversity index with total state Medicaid spending, FMAP, and per enrollee state Medicaid spending. Mann–Whitney U tests were used to compare census diversity index, total state Medicaid spending, FMAP, and per enrollee spending by political party (Democrat versus Republican). Time points corresponding to Medicaid data were used to generate political party leadership variables. For example, the census diversity index data correspond to 2020, so we used the results of the 2016 presidential elections, as the winner of the 2016 election was in office during the census survey period. Medicaid spending data were sourced from 2022 and 2023, corresponding to the presidency of the 2020 election winner. At the gubernatorial level, data from the 2020, 2021, 2022, and 2023 governors were used, corresponding to their respective data points. For Senate representation, data from the 116th Congress (3 January 2019–3 January 2021) were used for the 2020 diversity index, while data from the 117th (3 January 2021–3 January 2023) and 118th (3 January 2023–3 January 2025) congress were used for Medicaid spending. Importantly, we made comparisons between the U.S.’s two major political parties. To simplify the analysis and interpretation, states with split Senate delegations (i.e., members from different political parties) were excluded in some analyses. Similarly, due to highly split House delegations, U.S. House of Representatives members were not a focus of this analysis.

We also considered the impact of the Affordable Care Act (ACA), which gave states the option to expand Medicaid coverage to nearly all adults with incomes up to 138% of the federal poverty level. However, not all states have adopted this expansion, leaving a coverage gap for many low-income individuals. As of the end of 2022, twelve states had not expanded Medicaid: Alabama, Florida, Georgia, Kansas, Mississippi, North Carolina, South Carolina, South Dakota, Tennessee, Texas, Wisconsin, and Wyoming. By the end of 2023, both South Dakota and North Carolina had implemented Medicaid expansion. Given that expansion funding could impact our results, we subsequently examined relationships of Medicaid spending by political party affiliation in a sensitivity analysis limited to states without expansions.

Finally, the most recent Kaiser Family Foundation data available on per enrollee Medicaid spending by eligibility category (e.g., adults, children, seniors, and disabled persons) was from 2021. Using these data, we performed an additional sensitivity analysis exploring whether categorical Medicaid spending varied by political party affiliation. All statistical analyses were conducted using R Version 4.4.1 (R Core Team, Vienna, Austria). *p*-values < 0.05 were considered statistically significant.

## 3. Results

**Figure 1:**
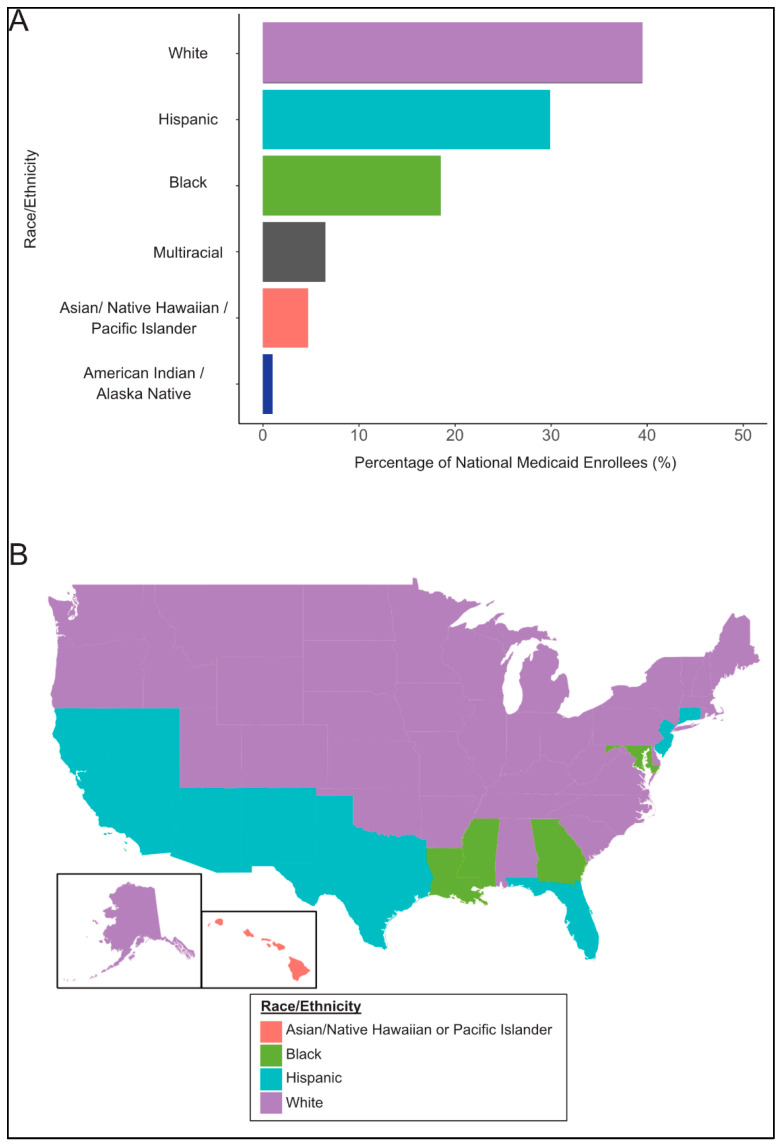
**Medicaid Enrollees by Race.** (**A**) presents the racial distribution of Medicaid enrollees nationally. (**B**) depicts a United States map with the geographical distribution of the single predominant race/ethnicity among Medicaid recipients in each of the 50 states and the District of Columbia. Graphs are based on 2023 Kaiser Family Foundation data from the Census Bureau’s American Community Survey.

Figure 1A presents a bar plot of the percentage of national Medicaid enrollees by race. Individuals identifying as White, Hispanic, and Black were the largest groups of enrollees in descending order. Figure 1B presents a U.S. map showing the single predominant race/ethnicity among Medicaid enrollees in each of the 50 states and the District of Columbia. Individuals identifying as Asian, Native Hawaiian, and Pacific Islander comprised the majority of Medicaid enrollees in one state (Hawaii). Individuals identifying as Black were the largest enrollee group in five states (District of Columbia, Georgia, Louisiana, Maryland, and Mississippi), while Hispanic individuals were the predominant group in eight states (Arizona, California, Connecticut, Florida, Nevada, New Jersey, New Mexico, and Texas). White individuals made up the majority of recipients in the remaining 37 states.

**Figure 2:**
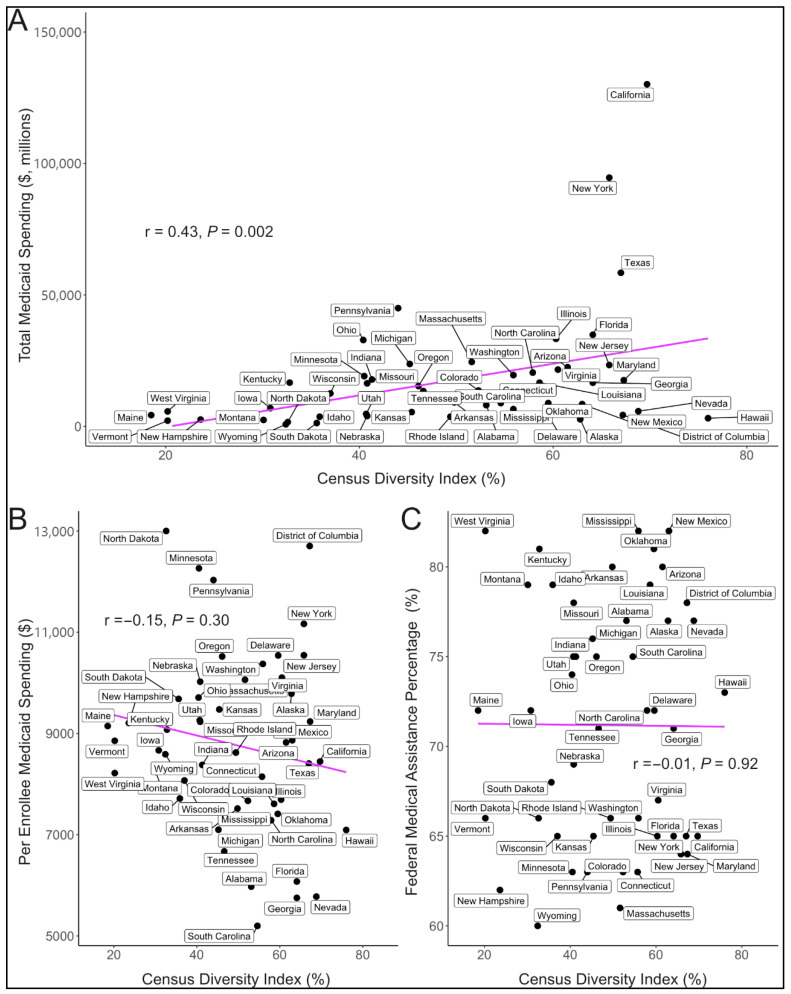
**Correlations of the diversity index with Medicaid Spending.** The figure depicts Spearman correlations for the 2020 diversity index with total Medicaid spending (**A**), per enrollee Medicaid spending (**B**), and the federal share of Medicaid spending (**C**) for each of the 50 states and the District of Columbia.

Figure 2 presents Spearman correlations of the census diversity index with total state Medicaid spending, per enrollee Medicaid spending, and FMAP. The census diversity index was moderately correlated with total state Medicaid spending (r = 0.43, *p* = 0.002, Figure 2A). Per enrollee Medicaid spending (r = −0.15, *p* = 0.30, Figure 2B) and FMAP (r = −0.01, *p* = 0.92, Figure 2C) were not correlated with the diversity index.

**Figure 3:**
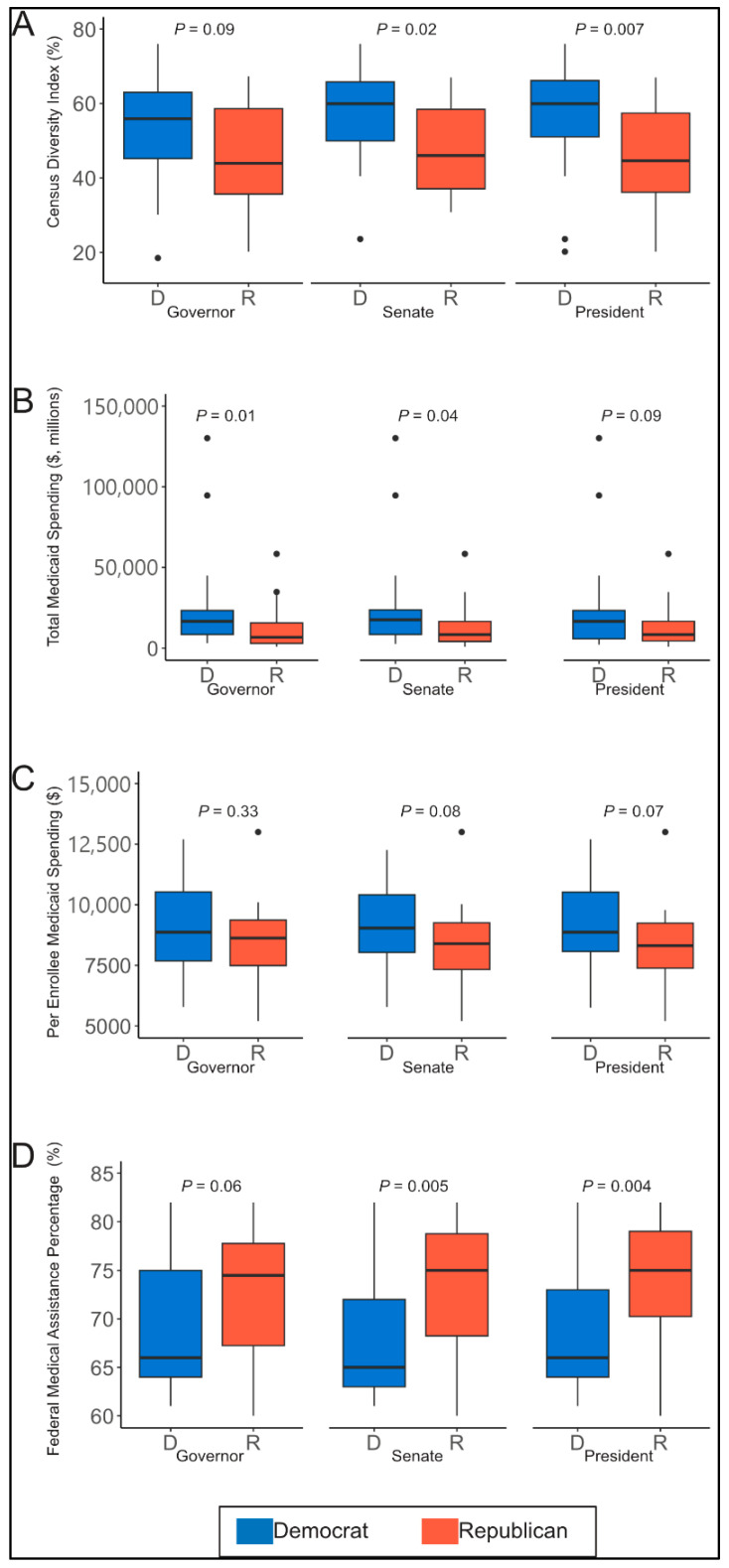
**Political party leadership patterns with diversity index and Medicaid spending.** The figure depicts patterns of state political party leadership (Democrat vs. Republican) with census diversity index (**A**), total Medicaid spending (**B**), per enrollee Medicaid spending (**C**), and the federal share of Medicaid spending (**D**).

Figure 3 presents relationships of the diversity index, total state Medicaid spending, per enrollee Medicaid spending, and FMAP by state-associated political party leadership. Democrats were found to represent more diverse states with statistically significant patterns in Senate and presidential representation (Figure 3A). In general, Democrat-represented states had greater total Medicaid spending (Figure 3B) and greater per enrollee Medicaid spending (Figure 3C) when compared to Republican-represented states. Nevertheless, these relationships were only statistically significant for total Medicaid spending at the levels of governor and Senate representation. While Democrat-represented states had higher total Medicaid spending, Republican-represented states demonstrated a statistically significant ~10% greater median share of Medicaid dollars from the federal government (Figure 3D). This pattern was statistically significant for Senate and presidential representation.

Among states that had not adopted Medicaid expansion, we found no statistically significant differences in per enrollee Medicaid spending by political party representation at either the gubernatorial (*p* = 0.37) or presidential (*p* = 0.61) levels. Notably, in the 117th Congress, there were no Democrat-led states at the Senate level that had not adopted Medicaid expansion. This changed in the 118th Congress when Georgia became the only Democrat-led state that had not adopted Medicaid expansion. Still, total Medicaid spending did not differ significantly by party at the gubernatorial (*p* = 0.53), presidential (*p* = 0.61), or Senate (*p* = 0.73) levels. FMAP spending also showed no significant differences by political affiliation at the gubernatorial (*p* = 0.34), presidential (*p* = 0.74), or Senate (*p* = 0.99) levels.

We found a statistically significant difference in per enrollee Medicaid spending for disabled persons at both the presidential (*p* = 0.02) and Senate (*p* = 0.02) levels, with Democrat-led states spending at least USD 4000 more per enrollee, on median, than Republican-led states. At the gubernatorial level, Democrat-led states spent approximately USD 2000 more per enrollee for disabled individuals, although this difference was not statistically significant (*p* = 0.14). No significant differences in per enrollee Medicaid spending by political affiliation were observed for adults at the gubernatorial (*p* = 0.64), presidential (*p* = 0.91), or Senate (*p* = 0.99) levels; for children at the gubernatorial (*p* = 0.88), presidential (*p* = 0.79), or Senate (*p* = 0.95) levels; or for seniors at the gubernatorial (*p* = 0.51), presidential (*p* = 0.12), or Senate (*p* = 0.16) levels.

## 4. Discussion

In this cross-sectional descriptive analysis of state Medicaid spending, we found that individuals who reported being White were the single predominant race of Medicaid beneficiaries in 37 states, followed by Hispanic individuals in eight states, Black individuals in five states, and Asian, Native Hawaiian, and Pacific Islander individuals in one state. Individuals identifying as White were also the leading group of Medicaid enrollees nationally. We observed strong correlations of the 2020 census diversity index with total Medicaid spending, but not the federal share of Medicaid spending or per enrollee Medicaid spending. Continued research will be key to understanding these differences. Lastly, we observed that Democrats more often represent states that are more diverse and have higher total Medicaid spending, but Republicans more often represent states that receive a higher share of federal funding to support their Medicaid spending. Together, our findings suggest that Medicaid impacts a diverse group of Americans and would impact constituents of both major political parties in a significant way.

We did observe some geographical trends in the location of the predominant single race of Medicaid enrollees by state and a moderate correlation of diversity with total Medicaid spending. This correlation is likely driven by state population, given that diversity was not correlated with per enrollee spending. We also found no relationship of diversity with the federal share of Medicaid spending. Together, we took these results to suggest that Medicaid spending impacts all racial groups.

Our Medicaid spending findings suggesting that the states represented by Democrats and Republicans are impacted differently are notable. One reason why Democrat-represented states may have greater total Medicaid spending when compared to Republican-represented states is because on average the populations of Democrat-represented states—particularly on the Senate and gubernatorial level—are greater. As such, when population size is considered (i.e., via per enrollee spending), significant differences between the political parties are no longer observed. The difference in the federal share of Medicaid spending is likely partially due to differences in state income levels. Republican-represented states receive a greater percentage of federal funds for Medicaid because these states often have higher rates of poverty, and income is factored into how federal funds are allocated [17]. Although these patterns appear straightforward, it is important to highlight that they are not static. Recent census data suggests that traditionally Republican-led states are growing faster in population than traditionally Democrat-led states [18]. Hence, metrics like total Medicaid spending could potentially be more of an issue for future Republican leaders.

We examined party leadership trends at three levels—Senate, gubernatorial, and presidential—as an indirect way of assessing which representatives may be most effective to target for advocacy. Interestingly, our strongest relationships were observed with Senate leadership. We observed relationships that were intermediate in strength at the level of president and governors. Together, these results suggest that engaging local stakeholders as well as policymakers who spend time in Washington, DC, may be the most effective strategy. Local stakeholders are particularly important as Medicaid is a federal-state partnership, and states have some latitude in deciding what services are available to beneficiaries. The cost-sharing structure, when paired with the existing state-level variability in eligibility, may mean that when faced with cuts to federal Medicaid dollars, states with higher FMAPs (i.e., relying more on federal assistance) may be more likely to implement cost-saving measures that could limit or cut services for present recipients. Local representatives not included in this study (e.g., state congressional representatives) will have an important say on how states address restrictions in funding [19,20]. Furthermore, our findings of significantly greater per enrollee spending for disabled persons in Democrat-led states, particularly when viewed through the lens of presidential and Senate leadership and, to a lesser extent, gubernatorial leadership, offer a compelling example of how Medicaid policy can reflect political priorities. This group-specific insight highlights an opportunity not only to advocate for Medicaid more broadly but also to champion the needs of a particularly vulnerable population. Such specificity may prove valuable in identifying targeted policy solutions and fostering areas of bipartisan collaboration.

This analysis does contain some important limitations. First, this is a cross-sectional analysis that cannot speculate on longitudinal relationships that are important for health insurance relationships. Secondly, the analysis uses publicly available data and is subject to any assumptions or limitations present in the original data sources. For instance, we found some conflicting data regarding the proportions of race/ethnicity of Medicaid enrollees for some states, including Arizona, Connecticut, Florida, Nevada, New Jersey, and New Mexico. These were states that we classified as having the largest fraction of enrol-lees being Hispanic. Still, we believe the overall message of the paper remains unchanged. Similarly, the diversity index is simply one measure of diversity, and other measures of diversity could produce different results. Nevertheless, we elected to use the diversity index because it was developed in a robust manner and could be easily applied to state-level data. Moreover, we are confident in all of our results, as they demonstrate similar trends present in other published literature. Third, we did not focus on the U.S. House of Representatives, given that delegations are often split between the political parties. Still, a prior published analysis of congressional districts demonstrates that at least 20% of the population in half of Republican and Democratic districts is enrolled in Medicaid [21]. This further supports the conclusions of our analysis that changes to the Medicaid program would affect many Americans living in areas represented by both major political parties. Finally, this analysis focuses on America’s two largest political parties but recognizes that other smaller parties can play a role in these important conversations nationally, but especially at local and regional levels.

## 5. Conclusions

In conclusion, our findings suggest that Medicaid plays an important role in supporting Americans across racial, geographic, and political lines, highlighting its broad and unifying impact as a public health safety net. Still, it is reasonable to recognize that Medicaid, like all human-designed programs, has limitations and areas for improvement. As such, conversations about reform and identifying areas where the program can be improved should continue. Nevertheless, it is important that human health remain at the center of these ongoing conversations and that any proposed changes are thoroughly vetted for intended and unintended consequences, agreed upon broadly, and implemented in a truly thoughtful manner. Only through such a careful process can we minimize harming Americans that direly rely on this important lifeline.

## Data Availability

All data is publicly available, and citations are provided within the manuscript and in the references section.
